# Toxicity of Naphthalene and Benzene on *Tribollium castaneum* Herbst

**DOI:** 10.3390/ijerph14060667

**Published:** 2017-06-21

**Authors:** Nerlis Pajaro-Castro, Karina Caballero-Gallardo, Jesus Olivero-Verbel

**Affiliations:** 1 Environmental and Computational Chemistry Group, Campus of Zaragocilla, School of Pharmaceutical Sciences, University of Cartagena, Cartagena 130001, Colombia; npajaroc@unicartagena.edu.co (N.P.-C.); kcaballerog@unicartagena.edu.co (K.C.-G.); 2 Medical and Pharmaceutical Sciences Group, School of Health Sciences, Department of Medicine, University of Sucre, Sincelejo 700003, Colombia

**Keywords:** naphthalene, naphthalin, benzene, mortality, gene expression, abnormalities

## Abstract

Naphthalene and benzene are widely-used volatile organic compounds. The aim of this research was to examine the toxicological effects of naphthalene and benzene against *Tribolium castaneum* as an animal model. Adult insects were exposed to these aromatic compounds to assess mortality after 4–48 h of exposure. The lethal concentration 50 (LC_50_) for naphthalene, naphthalin, and benzene were 63.6 µL/L, 20.0 µL/L, and 115.9 µL/L in air, respectively. Real-time polymerase chain reaction (PCR) analysis revealed expression changes in genes related to oxidative stress and metabolism [Glutathione S-Transferase (Gst), and Cytochrome P450 6BQ8 (Cyp6bq8)]; reproduction and metamorphosis [Hormone receptor in 39-like protein (Hr39), Ecdysone receptor: (Ecr), and Chitin synthase 2 (Chs2)]; and neurotransmission [Histamine-gated chloride channel 2 (Hiscl2)] in insects exposed for 4 h to 70.2 µL/L naphthalene. Adults exposed to benzene (80 µL/L; 4 h) overexpressed genes related to neurotransmission [GABA-gated anion channel (Rdl), Hiscl2, and GABA-gated ion channel (Grd)]; reproduction and metamorphosis [Ultraspiracle nuclear receptor (USP), Ecr; and Hr39]; and development (Chs2). The data presented here provides evidence that naphthalene and benzene inhalation are able to induce alterations on reproduction, development, metamorphosis, oxidative stress, metabolism, neurotransmission, and death of the insect.

## 1. Introduction

Of all the volatile organic compounds, benzene and naphthalene are amongst the most widely-used in diverse chemical, industrial, and commercial processes, as well as in combustion and evaporation of gasoline, and they are constituents of several commercial products such as cleaning fluids, paints, and glues. Naphthalene is a bicyclic aromatic compound used as the starting material for the synthesis of other compounds, as a moth repellent (naphthalin), soil fumigant, and lavatory deodorant [1]. Because of their low cost, naphthalin (Nap balls) are often used illegally for sale in the market, however, they are known to be harmful to humans [2]. Most exposure occurs through low dose chronic inhalation, dermal contact, or ingestion through the food chain [1]. These compounds are ‘high production volume’ chemicals (they are consumed in excess of one million pounds per year) used as industrial plasticizers, and they are ubiquitous environmental pollutants. The National Toxicology Program (NTP) and the International Association for Research on Carcinogens (IARC) classify naphthalene as rodent carcinogens that are “reasonably anticipated to be human carcinogens.” These chemicals do not seem to cause DNA damage in most assays, but their toxicity arises primarily in the metabolic process, and their nongenotoxic mechanisms of action are not yet known [3,4].

On the other hand, benzene is fairly soluble in water and is removed from the atmosphere in rain. The primary routes of exposure to this chemical are inhalation of contaminants, especially in the areas with high traffic, and consumption of contaminated drinking water [5]. A number of earlier studies reported the presence of benzene or benzene mixtures in varying concentrations in different occupational environments [6]. Chronic exposure to this chemical causes a reduction in the number of red blood cells and aplastic anemia, effects on reproduction and fetal development in animal tests, and increased incidence of leukemia in humans. Therefore, the Environmental Protection Agency (EPA) has classified this compound as a carcinogen by all routes of exposure in Group A [7]. The exposure to these non-oxygenated aromatic hydrocarbons is of great concern because of their toxicity and carcinogenicity, even at low concentrations. Contact with these substances comes from occupational exposure as well as from deliberate inhalation [6].

The present study deals with the effects of benzene and naphthalene on several biochemical markers of toxicity in *Tribolium Castaneum* (*T. castaneum*), as an alternative screening model to the use of large animals, with few ethical concerns. This insect has been employed as a research organism in several types of studies, including genetics and agriculture, especially in the search for new insecticides and in toxicity studies [8,9,10,11]. It presents many advantages, as it is easy to cultivate and handle, has a short generation time and large brood sizes, and its genome has been completely sequenced [10,11,12]. In fact, studies published in the last few decades suggest that invertebrate trials can be utilized as the first screening methods for assessing the lethal toxicity of new chemicals to mammals [13].

## 2. Materials and Methods

### 2.1. Beetle Cultures

*T. castaneum* specimens were taken from a stock colony maintained in the laboratory. Insects were fed a diet of ground oatmeal as well as oatmeal flakes (70:30) at 26 ± 2 °C, 70 to 85% relative humidity and a 10:14 light:dark photoperiod. Two- to four-week-old healthy adults were randomly selected for bioassays [14,15].

### 2.2. Mortality Assessments

Exposure of adult insects at different concentrations of these volatile compounds (0, 40, 80, 120, 160, and 240 µL/L air benzene and 0, 0.9, 4.4, 8.8, 17.5, 26.3, 35.1, 70.2, 105.3, 140.4, and 175.4 µL/L air naphthalene and naphthalin), were placed onto filter paper disks with a diameter of 1.74 cm, and then placed on the underside of the screw cap of a glass vial of 25 mL (8.1 × 2.86 cm). Ten *T. castaneum* specimens were placed into each vial without media before the cap was screwed tightly and the lid was sealed with parafilm [15]. For comparison purposes, a set of controls without compounds was maintained. The number of dead insects was counted at 4, 8, 24 and 48 h after treatment [16,17,18,19]. The criterion for mortality was the absence of movement in the limbs or antennae. The lethal concentration 50 (LC_50_) was calculated using Probit analysis. Since no mortality was observed in the controls, the corrections of treatment mortalities by Abbott’s formula [20] were not necessary. Terpinen-4-ol was used as a positive control, since this chemical is highly toxic to *T. castaneum* [18].

### 2.3. Analysis of Naphthalin Balls by Gas Chromatography-Mass Spectrometry (GC-MS)

The chromatographic study was performed using Agilent Technologies 7890A GC (Agilent Technologies Inc., Santa Clara, CA, USA) coupled to an Agilent Technologies MSD 5975 selective mass detector (Agilent Technologies Inc., Santa Clara, CA, USA), equipped with a DB-1MS capillary column (Agilent Technologies Inc., Santa Clara, CA, USA); 30 m long, 0.25 μm i.d., 0.25 μm film thickness, a split/splitless injector port (split), and an automatic Agilent 4513A injector (Agilent Technologies Inc., Santa Clara, CA, USA). The oven temperature was set at 80 °C for 1 min, and increased 5 °C/min up to 120 °C for 2 min. It was then raised 20 °C/min up to 150 °C for 2 min. Helium was used as a carrier gas with 1.2 mL/min linear velocity. The temperature of the split/splitless inlet, transfer line, and ion source were 250 °C, 280 °C, and 230 °C, respectively. The mass detector was operated in the electron ionization mode at 70 eV. Mass spectra and the total ion chromatograms were obtained by automatic scanning of a mass range (*m*/*z*) 30–700. The components were identified by comparing the mass spectrum determined for each chromatographic peak with those available from the Nist08 MS library, and quantified using their relative peak areas.

### 2.4. Analysis of T. Castaneum by GC-MS

Ten healthy insects, unexposed or exposed to the highest concentration of mothballs (175.4 µL/L, 48 h) were macerated with 1.5 mL of methanol. The obtained extracts were filtered twice with glass fiber and then placed in a vial for GC-MS analysis. The chromatographic study was performed as described for the analysis of naphthalin.

### 2.5. Gene Expression Assays

Treated and control whole adults were flash frozen in liquid nitrogen, and the total RNA was extracted using the RNeasy^®^ Mini Kit (Qiagen, Redwood City, CA, USA) according to the manufacturer’s instructions. The concentration of RNA was determined by spectrophotometry (A260) using a NanoDrop 2000 Spectrophotometer (Thermo Fisher Scientific, Waltham, MA, USA). Agarose (1.2%) gel electrophoresis was used to verify the quality of the RNA. Subsequently, cDNA was synthesized from 1.5 µg total RNA using the QuantiTect^®^ Reverse Transcription Kit (QiagenInc., Valencia, CA, USA). The resultant cDNA was used as the template in a 20-μL PCR reaction containing 10 pmol each of forward and reverse gene-specific primer. Real-time polymerase chain reaction (RT-PCR) was conducted on a StepOne^®^Plus (Applied Biosystems, Foster City, CA, USA). These reactions were carried out in MicroAmp optical 96-well reaction plates (Applied Biosystems, Foster City, CA, USA) utilizing Maxima SYBR Green/ROX qPCR Master Mix (Thermo Fisher Scientific, Waltham, MA, USA). The amplification was performed under the following conditions: 95 °C for 15 min to activate the DNA polymerase, then 40 cycles of 94 °C for 10 s, 50–60 °C for 30 s, and 72 °C for 30 s. In total, 12 genes were analyzed (Table S1), including Glucl (Glutamate-gated chroride channel), Rdl (GABA-gated anion channel splice variant 3a6a), Grd (GABA-gated ion channel), Hiscl2 (Histamine-gated chloride channel), Ache1 (Acetylcholinesterase 1), Sod (Cu/Zn-Superoxide Dismutase), Gst (Glutathione S-Transferase), Cyp6bq8 (Cytochrome P450 6BQ8), Chs2 (Chitin synthase 2), Hr39 (Hormone receptor in 39-like protein), Usp (Ultraspiracle nuclear receptor), and Ecr (Ecdysone receptor) [21,22,23]. Changes in gene expression were determined using ribosomal protein 18 (Rps18) and ribosomal protein 49 (Rp49) as housekeeping genes [24], and the comparative CT (ΔΔCT) method was utilized to determine the relative target quantity. The CT is defined as the PCR cycle at which the fluorescent signal of the reporter dye crosses an arbitrarily placed threshold. All experiments were run in duplicate and negative controls contained no cDNA [25,26,27].

### 2.6. Statistical Analysis

Probit analysis was employed to calculate LC_50_. Analysis of variance (ANOVA) with Tukey post-test was used to compare all groups (treated and control) at different time intervals. The data are presented as means ± SE and the differences between means are considered to be significant at *p* ≤ 0.05. GraphPad InStat 3.05 (GraphPad Software, La Jolla, CA, USA) was used for data analysis.

## 3. Results

### 3.1. Mortality Assessment

**Figure 1:**
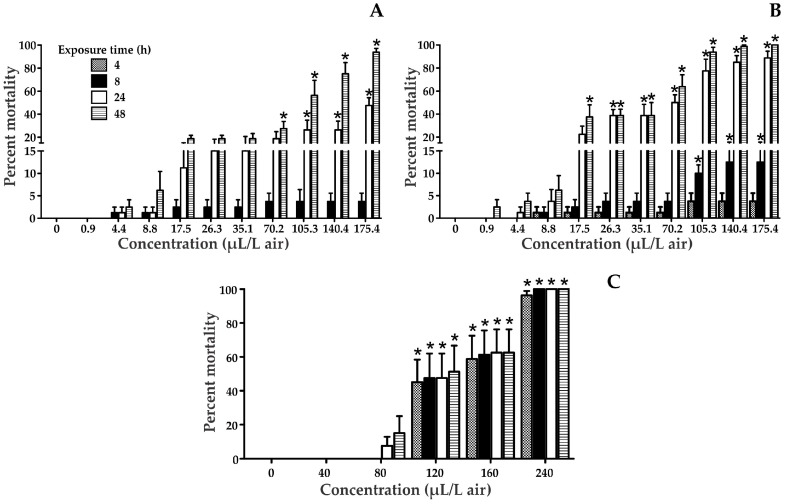
*Tribolium castaneum* mortality at 4, 8, 24, and 48 h after exposure to various solvents: Naphthalene (**a**); Naphthalin (**b**); Benzene (**c**). * Statistically significant compared to the control.

The Median Lethal Concentration (LC_50_) of naphthalene, naphthalin, and benzene were determined in *T. castaneum* adults exposed for 4, 8, 24 and 48 h (Figure 1 and Table 1). The results showed that naphthalin is highly toxic to *T. castaneum*. In all treatments, an increase in the percentage of mortality was observed according to the concentration and exposure time. In the case of benzene, significant differences compared to the control were detected at concentrations greater than 120 µL/L in air, and all times of exposure, while for naphthalin it was significant in the majority of cases in the longest exposure times. In addition, naphthalene showed significant differences from the control at the highest tested concentrations (105.3, 140.4, and 175.4 µL/L in air).

### 3.2. Gene Expression Analysis

**Figure 2:**
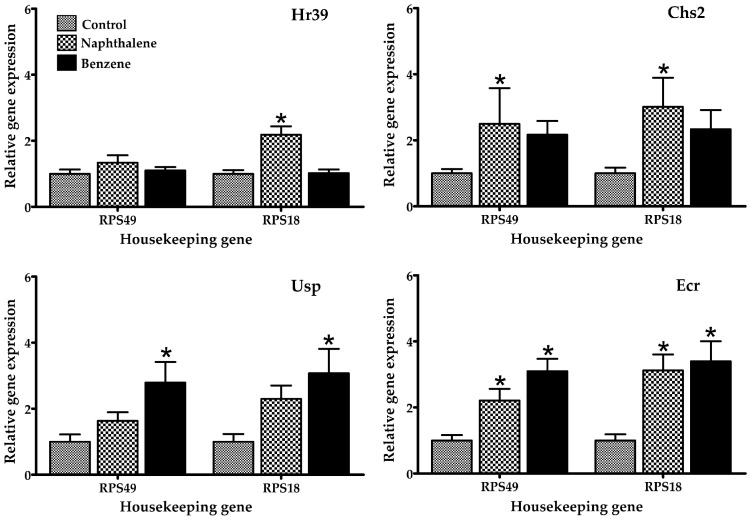
Relative mRNA expression of genes related to reproduction, development, and metamorphosis in adult *T. castaneum* after 4 h of exposure to naphthalene and benzene. Expression was normalized against Rps49 and Rps18 (housekeeping genes). * Significant difference (*p* < 0.05) when compared to the control.

**Figure 3:**
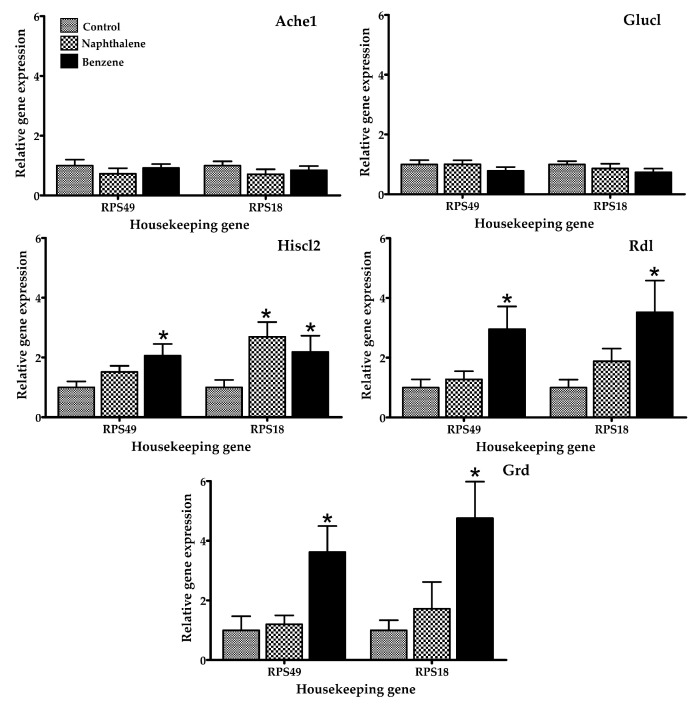
Relative mRNA expression of genes related to neurotransmission in adult *T. castaneum* after 4 h of exposure to naphthalene and benzene. Expression was normalized against Rps49 and Rps18 (housekeeping genes). * Significant difference (*p* < 0.05) when compared to the control.

**Figure 4:**
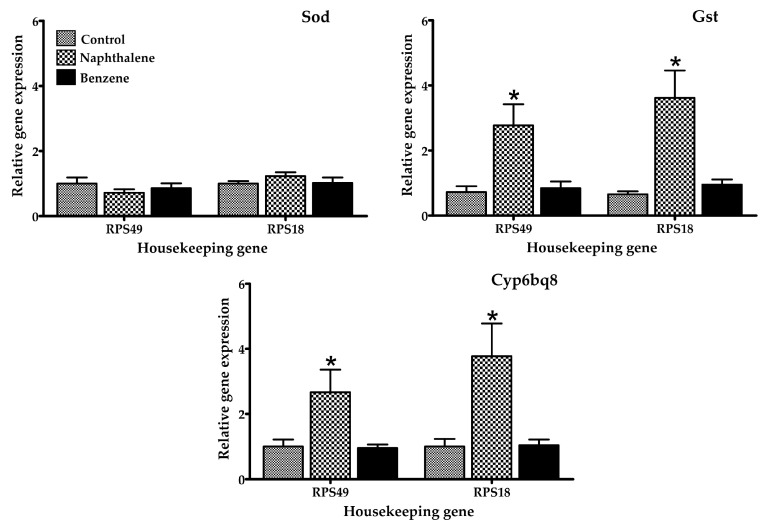
Relative mRNA expression of genes related to metabolic detoxification and oxidative stress in adult *T. castaneum* after 4 h of exposure to naphthalene and benzene. Expression was normalized against Rps49 and Rps18 (housekeeping genes). * Significant difference (*p* < 0.05) when compared to the control.

Several gene expression markers were evaluated in insects treated with a low toxicity concentration of naphthalene (70.2 µL/L) and benzene (80 µL/L air), concentrations at which 100% of insects were still alive after 4 h of exposure. The mRNA expression of selected genes in *T. castaneum* exposed to naphthalene and benzene is presented in Figure 2, Figure 3 and Figure 4. Insects in contact with naphthalene exhibited overexpression of Gst and Cyp6bq8, metabolic detoxification markers; Hr39, a sperm production regulator; EcR, important for metamorphosis; Chs2, involved in development by reducing the content of chitin; and Hiscl2, critical for neurotransmission. Moreover, benzene also affected pathways involving neurotransmission (Rdl, Hiscl2 and Grd); development (Chs2); and metamorphosis (Ecr and Usp). Although some other genes changed their expression patterns with exposure, such as Grd and Rdl, no significant differences were detected. Interestingly, the expression trend observed in red flour beetles exposed to naphthalene and benzene varied from gene to gene, suggesting specific types of responses in this organism.

## 4. Discussion

According to the lethality results presented in Table 1, the LC_50_ value for the positive control was similar to that reported in the literature [18]. In all cases, the chemicals examined followed a clear concentration-response relationship, with significant differences between concentrations at a particular time.

Naphthalin balls were more toxic to insects than naphthalene (Figure 1). It should be pointed out that although these balls contained mostly naphthalene, while the presence of benzo[b]thiophene (identified by gas chromatography/mass spectrometry, see Figure S1), may modify its toxicity by eliciting distinct biochemical interactions with cellular targets. On the other hand, insects unexposed and exposed to mothballs were analyzed by GC-MS, aiming to identify the presence of this chemical or its metabolites in the second group (Table S2). None of these chemicals was identified, perhaps because the concentration of the compound in the insect was low, or it was not efficiently bioaccumulated during exposure, as a result of the high volatility of naphthalene [27,28]. However, there was a change in the relative composition of extracted compounds in exposed insects, compared to those unexposed; this suggests that naphthalene changes the metabolome of the organism.

The adverse health effects of direct or indirect exposure to volatile aromatic compounds have been widely described in the literature [1,28]. Naphthalene is a white solid that exhibits a typical odor at room temperature, also known as naphthene, naphthalin, tar camphor, aldocarbon, or mothballs [29]. This chemical has been reported to cause toxicity in several animal species, such as dogs, rats, mice, rabbits, and humans [30], producing an increase in incidence and severity of olfactory epithelial metaplasia, chronic inflammation and hyperplasia of respiratory epithelium in the nose, and chronic inflammation in the lungs [1,31], effects that vary depending upon dose, route of exposure, and species involved [1]. Naphthalene is considered moderately toxic according to the EPA by inhalation exposure [30]. Mice exposed to 2 ppm naphthalene vapors for 4 h exhibited damage to Clara cells in the proximal airways [30]. In the present study, naphthalene and naphthalin balls were toxic for *T. castaneum*, a Coleoptera insect with no reported effects from these chemicals. In fact, the insect mortality was observed at 4.4 µL/L in air (1%) after 8 h of exposure and at 8.8 µL/L (1%) after 4 h exposure, respectively.

In the case of benzene, it is highly volatile, and exposure occurs mostly through inhalation [32]. Studies in animals through oral and inhalation exposure have demonstrated neurological, immunological, and hematological effects [33], as well as low inhalation toxicity in rats, mice, rabbits, and guinea pigs, moderate acute toxicity from ingestion, and low or moderate acute toxicity from dermal exposure [33]. In the present study, benzene generated toxic effects on insects exposed for 4 h at 120 µL/L in air (45%).

The effects of naphthalene and benzene are well-known in humans and rodents. Nevertheless, there is little data for other organisms. Mice exposure to naphthalene induces the activation of cytochrome p450, glutathione depletion, lipid peroxidation, DNA fragmentation, and the production of reactive oxygen species as superoxide anion and hydroxyl radical [1,34]. Naphthalene readily diffuses through tissues such as gills, and has been shown to bioaccumulate in various tissues in numerous aquatic invertebrate species. Juvenile animals are particularly sensitive to naphthalene due to their smaller body size and higher diffusion rates. Naphthalene exposure was also found to significantly impact cytochrome P450 genes, which have been linked with response to naphthalene exposure in copepods and fish [35]. The mechanism of toxicity of naphthalene involves its bioactivation by cytochrome P450 enzymes, inducing acute cytotoxicity through repeated cycles of injury and repair [36] and causing damage in multiple organs, including the lung, liver, and kidney, by producing free radicals and triggering the immune response [37]. Naphthalene-induced respiratory toxicity is related to lipid peroxidation, disruptions of membrane components, and imbalanced energy supply [37]. When the mouse lung is under the attack of reactive naphthalene metabolites, glutathione-S-transferase catalyzes the conjugation of glutathione to electrophilic compounds and further metabolizes to water-soluble compounds that can be excreted in the urine. Studies have demonstrated that glutathione loss increases protein adduct formation and precedes injury [37]. In *T. castaneum*, naphthalene activates markers of oxidative stress and metabolism (Gst and Cyp6bq8), as reported by other studies for other organisms. This indicates that the insect is able to metabolize the naphthalene. In addition, gene expression of Gst was elevated; this gene is an indicator of oxidative stress. Several studies have shown the overexpression of Gst in organisms exposed to naphthalene [38,39], these findings agree with the results observed in this study. In fact, in mammals, this chemical is metabolized in the presence of cytochrome p-450 [29]. Also, some of the overexpressed genes were those related to reproduction (Hr39 and Ecr), confirming the toxic action of naphthalene in the reproduction of insects, a finding also reported in rats, mice, and rabbits [40]. These results confirm the toxicity of naphthalene at the insect’s cellular level, as also found in other organisms [35,37,40].

Insect species with sequenced genomes are being proposed as models to study the molecular basis of disease [38]. Interestingly, it has been demonstrated that *T. castaneum* sequences are 70% more similar to human than Drosophila genes [39]. In *T. castaneum*, benzene increased the expression of Rdl, Grd, Hiscl2, Chs2, Ecr, and Usp genes, with significant differences between groups (treated and control). Recent studies report that the chronic benzene exposure, even at levels below the current U.S. occupational standard, perturbs many genes, biological processes, and pathways [41]. Their effects include the generation of free radicals, which leads to oxidative stress, immune system dysfunction, and decreased immune surveillance [41], in addition to affecting pathways of biotransformation, glutathione synthesis, fatty acid and cholesterol metabolism, and others [42]. This compound is metabolized mainly by the enzyme cytochrome p-450; however, in this study no activation of this gene was observed, perhaps due to differences between mammals and non-mammals [43]. Interestingly, benzene exposure resulted in classic symptoms of central nervous system (CNS) depression [44], and possessed neurotoxic and behavioral effects [45]; in this study, the insects exposed to this chemical showed expression of Rdl, Hiscl2, and Grd genes, which are related to neurotransmission.

## 5. Conclusions

In summary, naphthalene and benzene were toxic to *T. castaneum*. Naphthalene provoked the activation of genes related to oxidative stress, metabolism, and reproduction. Benzene overexpressed genes related to neurotransmission and reproduction. *T. castaneum* may be used as an alternative animal model for toxicity testing, since it provides an accessible system for evaluating distinct signaling pathways, similar to those found in superior organisms, with minor ethical concerns.

## Figures and Tables

**Table 1:** Toxicity of naphthalene, naphthalin, and benzene on *Tribolium castaneum*.

	Naphthalene	Naphthalin	Benzene	Positive Control Terpinen-4-ol
Exposure Time (h)	LC50 (µL/L Air)			
	(95% CL) *			
4	>175.4	>175.4	158.9 (151.8–166.4) [9.7 ± 0.1; 22.5]	>240
8	>175.4	>175.4	134.3 (129.4–139.3) [12.1 ± 0.1; 23.9]	>240
24	>175.4	49.9 (44.0–56.4) [2.5 ± 0.1; 1.33]	118.6 (113.8–123.6) [10.8 ± 0.0; 12.9]	40.4 (25.4–64.2) [1.0 ± 0.1; 0.043]
48	63.6 (55.4–73.0) * [2.3 ± 0.0; 0.04] **	20.0 (17.4–22.9) [2.3 ± 0.1; 1.89]	115.9 (111.1–120.8) [10.6 ± 0.1; 4.62]	<40.0 [<37] ***

* (95% Confidence limits); ** [Slope ± SE; Chi-square]; *** Reported by Suthisut et al., 2011 [18].

## Supplementary Materials

The following are available online at www.mdpi.com/1660-4601/14/6/667/s1, Table S1: Primer sequences used for genes investigated by RT-PCR, Table S2: Compounds identified by GC/MS in *T. castaneum* unexposed or exposed to nap balls (naphthalin), Figure S1: Typical GC/MS chromatograms of nap balls (naphthalin).
