# Supplementary material for: Toxicity of Naphthalene and Benzene on Tribollium castaneum Herbst
Source: Int J Environ Res Public Health. 2017 Jun 21;14(6):667. doi: 10.3390/ijerph14060667 (PMC5486353; doi:10.3390/ijerph14060667)
Supplement: Supplementary file 1 [file ijerph-14-00667-s001.pdf]

Table S1. Primer sequences used for genes investigated by RT-PCR.

**Gene name**	*Gene Symbol*	*Entrez gene ID*	*Forward (5′-3′)*	*Reverse (5′-3′)*	*Amplicon Size* **(pb)**
			Target Genes		
Acetylcholinesterase	AchE1	HQ260968.1	CCGTTCGTCCCAGTCATTG	AGTAGTAGCCTTCTTCTGTGTTAG	121
GABA-gated anion channel splice variant 3a6a	RDL	NM_001114292.1	ACTTGGGCGACGTCAACATA	ACGTGAAATCCATCTGGACC	159
GABA-gated ion channel	GRD	NM_001114300.1	GGTCTCCTTCTGGCTGAACC	TGGACCACAGCGAACTGAAT	198
Glutamate-gated chloride channel	GluCl	NM_001114304.1	TGAATGGCACAGATGGTCCC	CCAGACTCGACTGGCTTCAG	194
Histamine-gated chloride channel 2	HisCl2	NM_001109951.1	TGGATGTCCAGTTGTTCGGT	TGTGGCTGAATAGGCAAGTCAT	176
Hormone receptor in 39-like protein	HR39	XR_043083.1	CGACCGTCGACTGTACAAAA	AGTCGACATGGAACGGAAAC	145
Ultraspiracle nuclear receptor	USP	NM_001114294.2	GATGCAAGCACAGGATGCTA	CCGACTTTATCCCTCGAACA	206
Ecdysone receptor	EcR	NM_001141918.1	GATGGATGGCGAAGATCAGT	ACTTCGCTGGAACATGCTTT	162
Chitin synthase 2	CHS2	AY295879.1	TCCTCCTAGTCATCTACTCG	TTATGCCTCCACGTCTGACC	2289
Cytochrome P450 6BQ8	CYP6BQ8	XM_970474.1	CTATCCCCAACACTTCCATTC	CTGCGGTCAGTCCAACTTTAC	237
Glutathione S-Transferase	GST	XM_964053.1	ATTCGATTGCCCGATATTTG	CGTTGAAGGTGTCCACAATG	96
Cu/Zn-Superoxide Dismutase	SOD	XM_963191.2	CCATGGAAAGGATCATGGAG	GGCTTCGATATTCCCCAAAT	70
			**Housekeeping genes**		
Ribosomal protein Rp49	Rp49	XM_964471.2	TGGCAAACTCAAACGCAACT	AGCGCCTACGAACCCTGTT	62
Ribosomal protein RpS18	RpS18	XM_968539.2	CGAAGAGGTCGAGAAAATCG	CGTGGTCTTGGTGTGTTGAC	235

Table S2. Compounds identified by GC/MS in *T. castaneum* unexposed or exposed to nap balls (naphthalin).

Peak	Compound	Retention time (min)	Area (%)	
			Unexposed	Exposed
1	1,3-bis(3-phenoxyphenoxy)benzene	1.50	**4.95**	**25.75**
2	14-Methylpentadecanoic acid	29.00	19.68	20.26
3	9,12-octadecadienoic acid methyl ester	32.04	**58.86**	**30.31**
4	Unidentified	36.09	0.79	9.45
5	1-Heptadecanamine	38.22	15.58	14.12
	Others	---	0.14	0.11

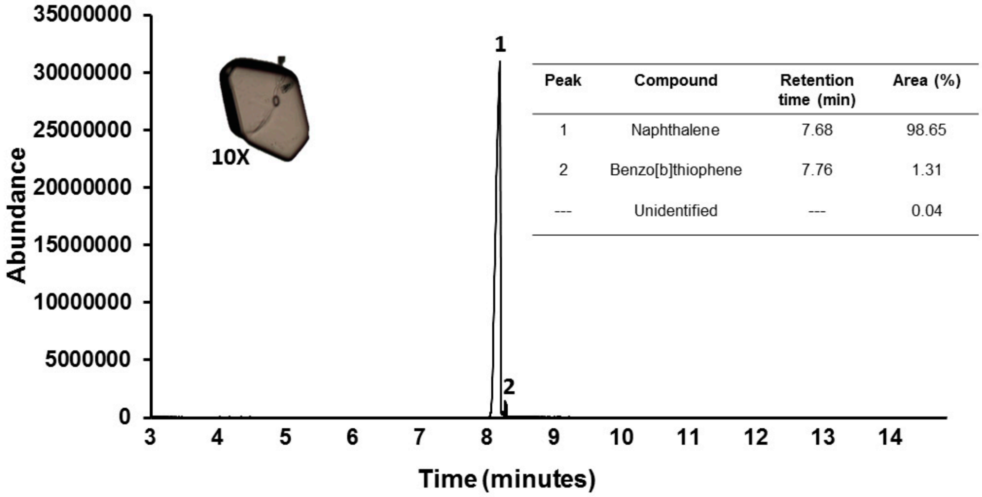

Peak	Compound	Retention time (min)	Area (%)
1	Naphthalene	7.68	98.65
2	Benzo[b]thiophene	7.76	1.31
---	Unidentified	---	0.04

Figure S1. Typical GC/MS chromatogram of nap balls (naphthalin).
